# Ileocolic Intussusception Due to Mucinous Adenocarcinoma in a Middle-Aged Man: A Rare Presentation

**DOI:** 10.7759/cureus.78136

**Published:** 2025-01-28

**Authors:** Ananya Johari, Samir Ahmad, Karthikeyan Selvaraj, Ramki Arunachalam Ganesh

**Affiliations:** 1 General Surgery, Sree Balaji Medical College and Hospital, Chennai, IND

**Keywords:** adult intestinal invagination, adult intussusception, cecal adenocarcinoma, ileocolic intussusception, ileocolic type

## Abstract

The invagination, or telescoping, of one segment of the colon into another distal segment is the hallmark of the medical disorder known as intussusception. The invaginated portion of the intestines may be propelled forward by peristaltic movements, which may result in bowel blockage, ischemia, and long-term necrosis. The precise cause of intussusception is ambiguous, particularly in cases with no clear lead point. Dysrhythmic contractions and lymphoid hyperplasia are nevertheless linked to the pathophysiology. A 54-year-old male patient arrived at our emergency department after experiencing abdominal pain that had been progressively increasing for the past three days. The patient had a history of previous appendectomy. It was reported that the pain was abrupt and severe and that it grew worse with each meal or drink. During the physical examination, abdominal distension, discomfort, central guarding, and a small palpable mass measuring 3 x 3 cm were identified. Contrast-enhanced CT scans revealed a 7 cm segment intussusception of the terminal ileum into the cecum and ascending colon. Furthermore, the cecum, mesentery, vasculature, and subsequent nodes were all involved in a significant amount of wall edema. During an emergency laparotomy, a terminal ileocolic intussusception was identified. A restricted segmental resection of the terminal ileum was conducted after the adhesiolysis. Subsequently, an end ileostomy was performed. Ileocolic obstructive intussusception is a rare adult condition caused by a mucinous adenocarcinoma. This case provides a unique perspective on the condition. Consequently, physicians must be vigilant for indications of obstructive intussusception in various colon regions that may suggest malignancy.

## Introduction

Intussusception is when the proximal bowel segment (intussusceptum) invaginates into the distal bowel segment (intussuscipiens). This may result in intestinal obstruction and reduced blood supply to the region. In rare cases, the intussusceptum may become necrotic, gangrenous, and strangulated, resulting in peritonitis or mortality. Intussusception in adults is rare, occurring in fewer than one in 1,300 abdominal surgeries. It is more commonly observed in children, with a ratio of approximately 20:1 compared to adults. In adults, intussusception accounts for about 1% of cases of small bowel obstruction, with tumors being the most frequent underlying cause [1]. Intussusception accounts for approximately 1% of all adult bowel obstructions [2]. In contrast to the idiopathic nature of intussusception in children, it is more frequently a result of an underlying condition in adults and is characterized by a lead point [3]. Intussusceptions result from malignant tumors in 65% to 87% of all adult cases, according to reports [3-5]. In adults, intussusception frequently manifests as nonspecific symptoms, including abdominal pain, nausea, diarrhea, and rectal hemorrhage. The classical triad of symptoms, which includes a sausage-shaped palpable mass, red currant jelly feces, and acute abdominal pain, is less frequently observed in adults [2,6]. Surgical resection is typically recommended due to the high incidence of intussusception in adults that is caused by malignancy [7]. We report a rare instance of colon carcinoma in a middle-aged male who presented with intestinal obstruction as a result of ileocolic intussusception.

## Case presentation

**Figure 1:**
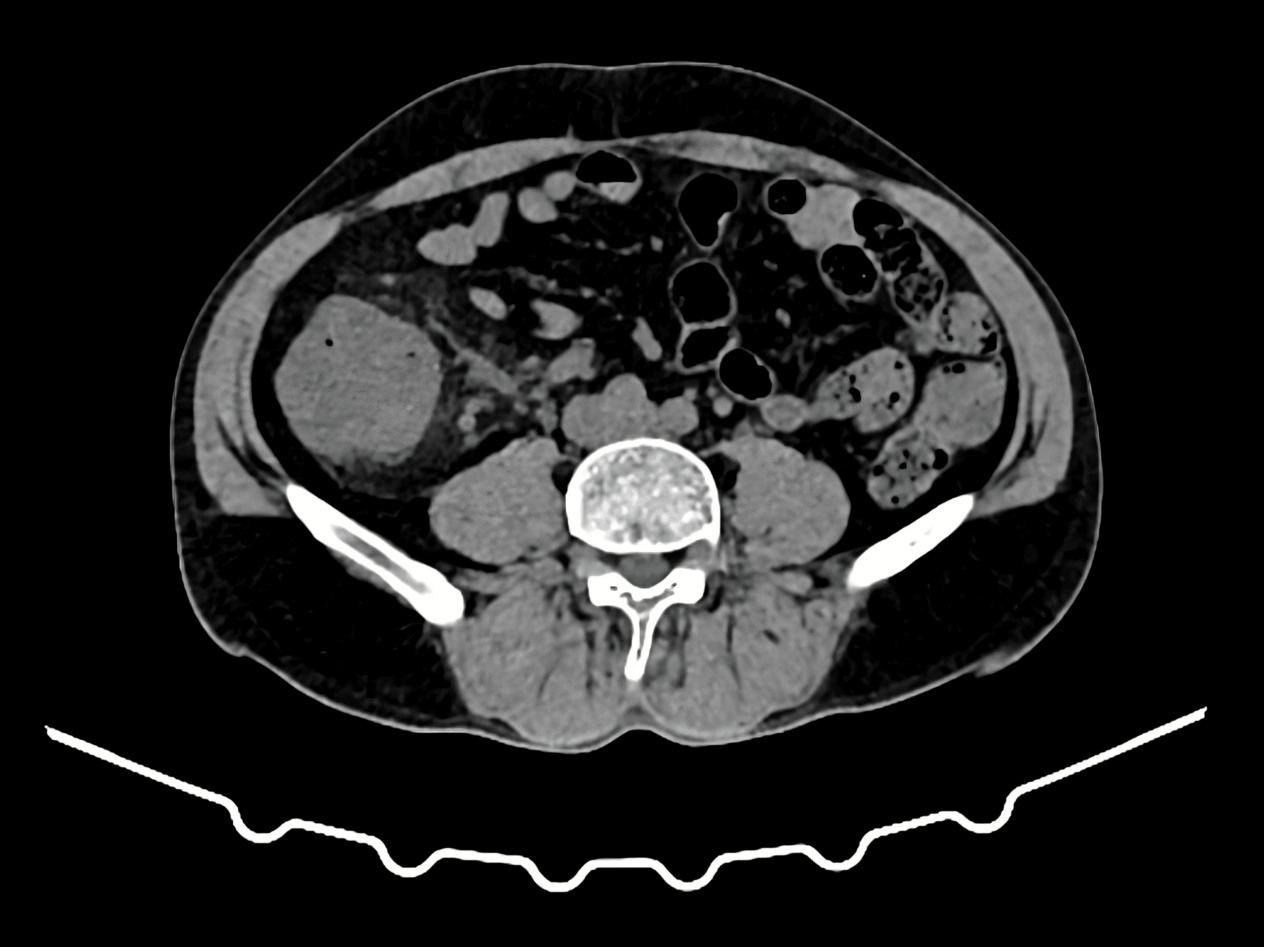
CECT scan revealed a 7 cm long segment intussusception of the terminal ileum into the caecum and ascending colon, with the mesentery and its vasculature CECT: contrast-enhanced computed tomography

A 54-year-old male presented with acute abdominal pain, notably in the area of the umbilicus. Additionally, he reported that his regurgitation was blood-stained and was not projectile or bilious. For one day, the patient was unable to expel feces. The patient had a history of decreased urinary output and had previously undergone an appendicectomy. The patient was afebrile and oriented during the examination. Central guarding, abdominal distension and tenderness, and a small palpable mass measuring 3 x 3 cm are all present in the abdomen. The digital examination was normal. The patient's bloodwork was unremarkable, and vitals were stable. A radiogram of the erect abdomen was obtained, which revealed a few air-fluid levels but no pneumoperitoneum. The telescoping of the terminal ileum into the colon and diffuse circumferential mural enlargement of the caecum and ascending colon were observed in the right lumbar region during initial ultrasonography of the abdomen. The contrast-enhanced CT (CECT) scans revealed a 7 cm long segment intussusception of the terminal ileum into the caecum and ascending colon, with the mesentery and its vasculature, nodes trailing along, and significant wall edema involving the caecum (Figure 1).

**Figure 2:**
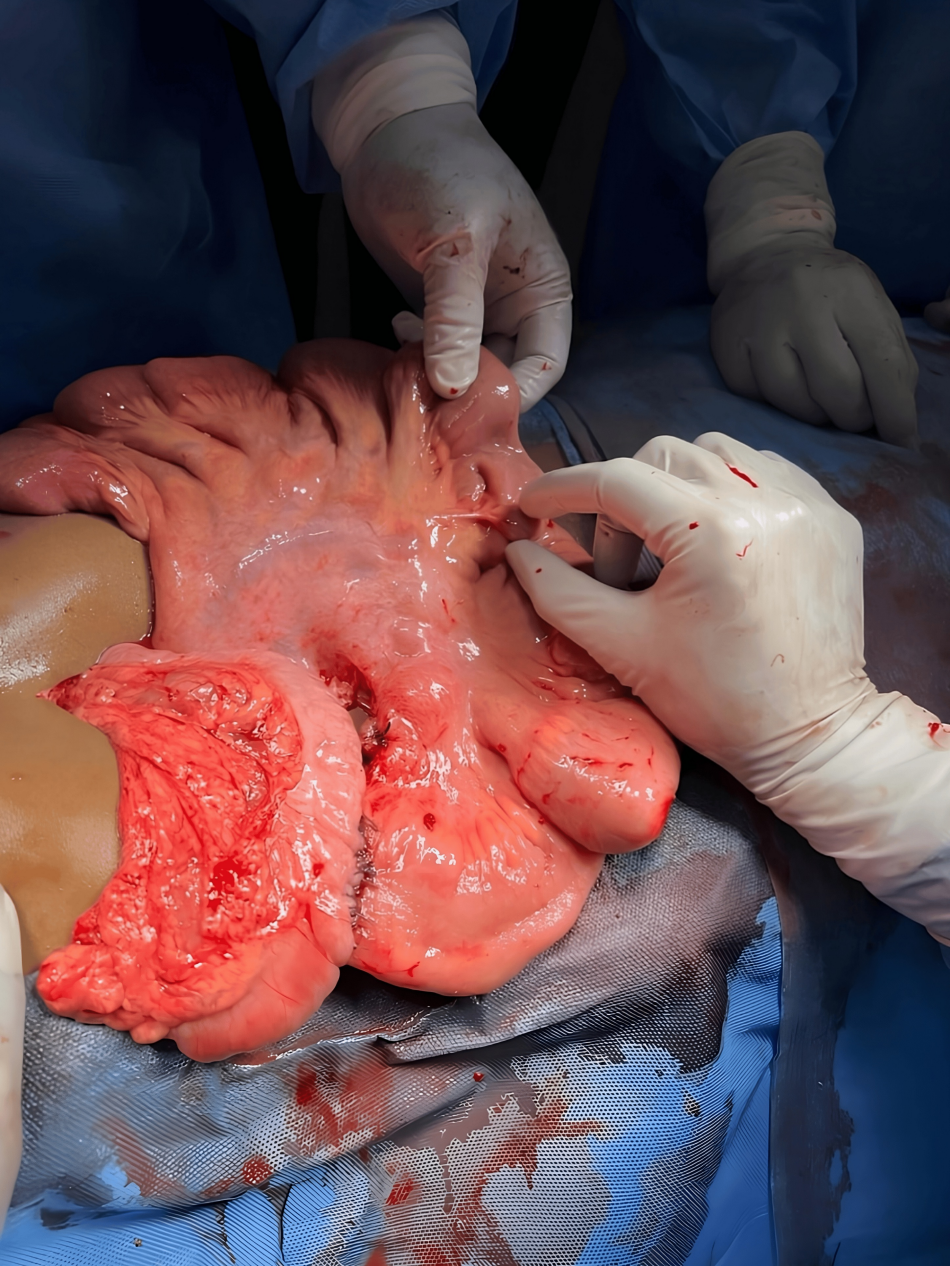
Adhesive band was observed in the right iliac fossa

Initially, the patient was treated conservatively with intravenous fluids and antibiotics, as well as Ryles tube decompression. An emergent laparotomy was performed after an ileocecal obstruction diagnosis was rendered. An adhesive band was observed in the right iliac fossa, and the small bowel was distended intraoperatively through gastric dilation (Figure 2).

**Figure 3:**
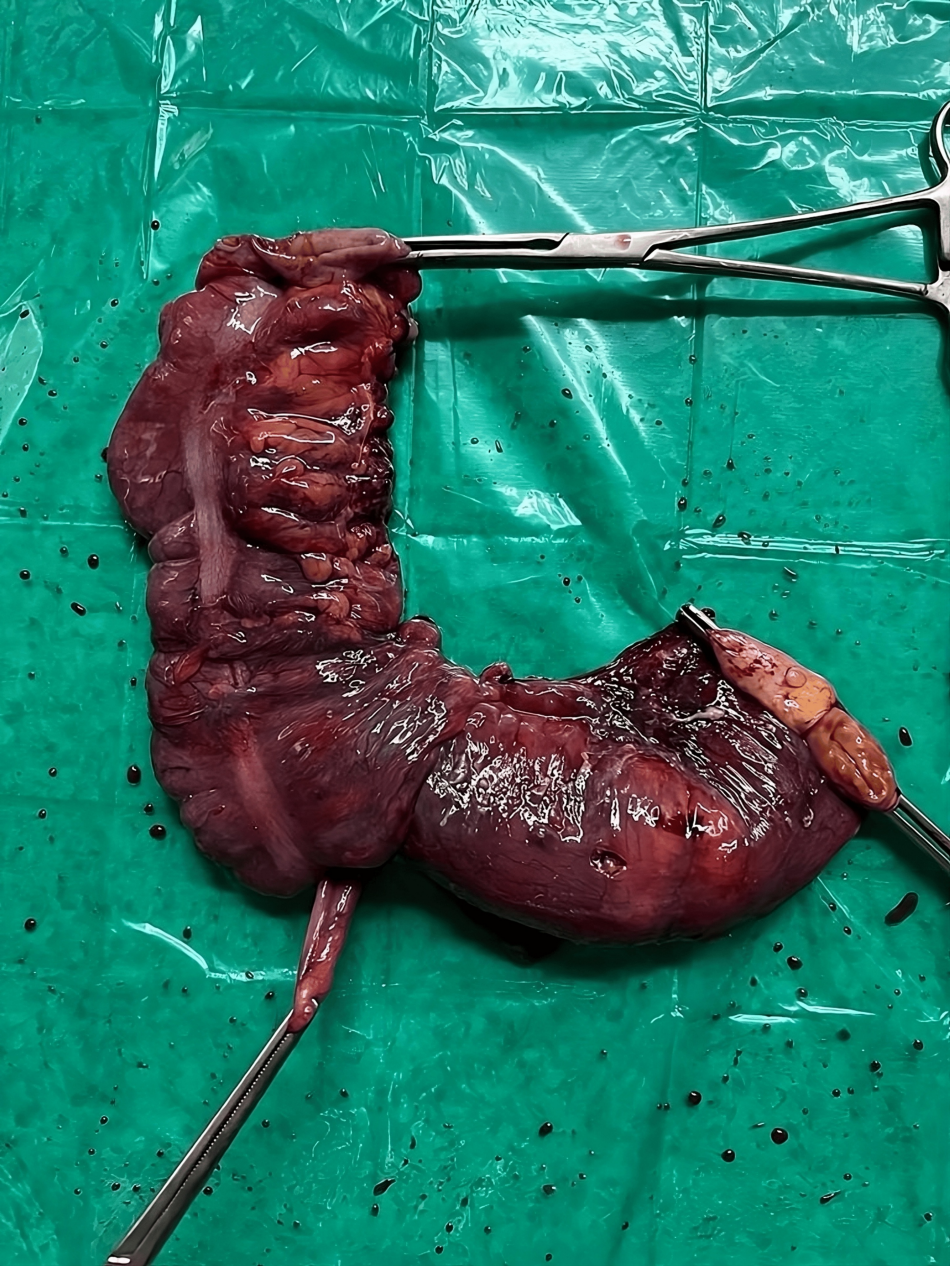
Surgically resected specimen of the terminal ileum, caecum, and ascending colon along with the mesentery

An ileocolic intussusception was present in the terminal ileum. Limited segmental resection of the terminal ileum, caecum, and ascending colon along the mesentery was performed after the adhesive band was released by digital dissection (Figure 3).

The end was closed, and the ascending colon and the right 5-7 cm of the transverse colon were resected. An ileostomy was conducted after the stab incision was made in the right iliac fossa, and 5-7 cm of the terminal ileum was resected. The patient's postoperative course was consistent with expectations. The histopathology of the resected specimen revealed an adenocarcinoma with mucin secretion. The patient was referred to a nearby oncology center for chemotherapy and radiation. An end-to-side anastomosis was performed, and an ileostomy closure was scheduled after six months. The postoperative period was uneventful, and bowel sounds were detected on the fourth postoperative day. A liquid diet was implemented. The catheter was removed on postoperative day 7, and a soft, solid diet was subsequently implemented.

## Discussion

Adult intussusception is a rare disorder. Diagnosing adult intussusception is difficult since the symptoms present at the time of presentation are wide and vague [2,6]. Contrary to the sudden and severe abdominal pain often seen in children, adults usually have varying episodes of abdominal pain. Additional prevalent symptoms in adults include hematochezia (blood in the stool), vertigo (dizziness), and vomiting [4,8]. Colonic intussusceptions are more prone to inducing bleeding than ileal intussusceptions. Only around 10% of adult cases exhibit detectable abdominal masses, which starkly contrasts with pediatric populations, where a palpable, sausage-shaped mass is often identified during physical exams [2,6]. The patient had symptoms of stomach discomfort, nausea, and vomiting, which are typical of enteric intussusception. Colonic intussusception may manifest with symptoms such as constipation, lower gastrointestinal hemorrhage, and weight loss. Intussusception is usually of unknown cause in children, but in adults, fewer than 10% of occurrences are attributed to unknown reasons [9]. Studies show that malignant tumors are responsible for up to 87% of occurrences of adult intussusception, with the specific percentage varying depending on the location of the lead point [3-5]. The majority of instances are attributed to benign tumors. The colon has the greatest cancer occurrence, but enteric intussusception is less common [10].

Less than half of adult intussusception cases are correctly identified prior to surgery [2]. Abdominal CT scans often reveal pseudokidney signals on coronal sections and target or doughnut signs on cross-sections [11,12]. Barium tests may also assist in the diagnostic procedure. A colonoscopy is indicated when CT scans show abnormalities in the colon [13]. There is ongoing discussion over the most effective strategies for addressing adult intussusception. The choice of treatment techniques is determined by factors such as the patient's clinical state, underlying etiology, suspicion of malignancy, and the location of the intussusception. However, the most prevalent therapy for adults, particularly for colonic intussusception, is surgical removal of the abnormal growth, mostly because of the significant likelihood of cancer. Occasionally, the lesion may undergo reduction prior to surgery. If there are no indications of cancerous growth and the gut is not experiencing reduced blood flow or is easily damaged, a surgical procedure to reduce enteric intussusception may be considered [4,9,14]. Typically, the outlook for adult patients with intussusception caused by nonmalignant causes is positive. Regrettably, the general survival chances within this group are diminished since a significant proportion of patients have intussusception as a result of malignant tumors. The death rate in instances of intussusception caused by a benign lesion is below 10% in adults. However, when malignancy is the underlying cause, the death rate surpasses 50% [2].

## Conclusions

Adult intussusception is a rare and difficult entity to diagnose for clinicians because of the nonspecific symptoms that accompany the condition. The most effective and minimally invasive diagnostic instrument is a CT scan of the abdomen. As there is a high likelihood of underlying malignancy, the preferred treatment for adults is operative resection without reduction.
